# Association Between Prenatal Exposure to Metals and Atopic Dermatitis Among Children Aged 4 Years in Taiwan

**DOI:** 10.1001/jamanetworkopen.2021.31327

**Published:** 2021-10-27

**Authors:** Tsung-Lin Tsai, Shu-Li Wang, Chia-Jung Hsieh, Hui-Ju Wen, Chin-Chi Kuo, Huei-Ju Liu, Chien-Wen Sun, Mei-Lien Chen, Ming-Tsang Wu

**Affiliations:** 1 National Institute of Environmental Health Sciences, National Health Research Institutes, Miaoli, Taiwan; 2 Department of Medical Research, China Medical University Hospital, China Medical University, Taichung, Taiwan; 3 Department of Healthcare Administration, Asia University, Taichung, Taiwan; 4 Graduate Institute of Life Science, National Defense Medical Center, Taipei, Taiwan; 5 Department of Safety, Health, and Environmental Engineering, National United University, Miaoli, Taiwan; 6 Department of Public Health, Tzu Chi University, Hualien, Taiwan; 7 Big Data Center, China Medical University Hospital, Taichung, Taiwan; 8 Division of Nephrology, Department of Internal Medicine, China Medical University Hospital, Taichung, Taiwan; 9 Institute of Environmental and Occupational Health Sciences, College of Medicine, National Yang Ming Chiao Tung University, Taipei, Taiwan; 10 Research Center for Environmental Medicine, Kaohsiung Medical University, Kaohsiung, Taiwan

## Abstract

**Importance:**

The prevalence of atopic dermatitis has substantially increased in recent decades, and atopic dermatitis could lead to allergic airway inflammation later in life. A previous study found that inorganic arsenic exposure was associated with allergic airway inflammation in children aged 8 to 14 years. However, the association between prenatal exposure to arsenic and other metals and the risk of atopic dermatitis among young children remains unknown.

**Objective:**

To assess the association between prenatal exposure to arsenic and other metals and the occurrence of atopic dermatitis in children at age 4 years.

**Design, Setting, and Participants:**

In total, 1152 pregnant women were enrolled in the original Taiwan Maternal and Infant Cohort Study (TMICS), a multicenter birth cohort study conducted at 9 hospitals in northern, central, southern, and eastern Taiwan from October 2012 to May 2015. Of those, 586 mothers and children aged 4 years participated in follow-up questionnaire interviews from August 2016 to January 2019. After excluding 216 participants with missing data, the final statistical analysis of follow-up data included 370 mother and child pairs from the central and eastern regions of Taiwan. Data were analyzed from February 2 to August 12, 2021.

**Exposures:**

Arsenic, cadmium, lead, cobalt, copper, nickel, thallium, and zinc during pregnancy.

**Main Outcomes and Measures:**

The outcome was parent-reported atopic dermatitis history among children aged 4 years. The presence of atopic dermatitis was defined as a positive response to the question, “Has your child ever had atopic dermatitis diagnosed by a physician?” During the initial TMICS study period, concentrations of arsenic, cadmium, lead, cobalt, copper, nickel, thallium, and zinc were measured in maternal urine during the third trimester of pregnancy using an inductively coupled plasma mass spectrometer. Estimated total inorganic arsenic exposure was calculated using a model that included data on both total arsenic and arsenic species (arsenite, arsenate, monomethylarsonate, and dimethylarsenate) obtained from a previous TMICS cohort.

**Results:**

Among 370 children included in the analysis, the mean (SD) age was 3.94 (0.59) years; 208 children (56.2%) were male, and 267 children (72.2%) were from the central region of Taiwan. A total of 110 children (29.7%) had atopic dermatitis at age 4 years. Maternal estimated total inorganic arsenic exposure during pregnancy was associated with increased odds of atopic dermatitis among children at age 4 years (odds ratio [OR], 2.42 [95% CI, 1.33-4.39] for every doubled increase of total inorganic arsenic) after adjusting for parental allergies, child’s sex, geographic area, maternal educational level, and exposure to tobacco smoke. Every increased unit in the weighted quantile sum index of maternal metal exposure was significantly associated with atopic dermatitis (OR, 1.63; 95% CI, 1.28-2.07). Arsenic (40.1%) and cadmium (20.5%) accounted for most of the metal mixture index.

**Conclusions and Relevance:**

This cohort study found that prenatal exposure to inorganic arsenic and coexposure to inorganic arsenic and cadmium were associated with a higher risk of atopic dermatitis in young children. These findings suggest that prevention of exposure to inorganic arsenic and cadmium during pregnancy may be helpful for the control of atopic dermatitis and other potential allergies in children.

## Key Points

**Question:**

Is prenatal exposure to arsenic and other metals associated with the occurrence of atopic dermatitis in young children?

**Findings:**

In this cohort study of 586 mother and child pairs, prenatal arsenic exposure was associated with approximately 2.4 times the occurrence of atopic dermatitis in children at age 4 years. In addition, coexposure to arsenic and cadmium was associated with increased odds of developing atopic dermatitis.

**Meaning:**

The results of this study suggest that prevention of inorganic arsenic and cadmium exposure among pregnant women could reduce the risk of atopic dermatitis and other allergic diseases in children.

## Teaser

This cohort study examines the association between prenatal exposure to arsenic and other metals and the development of atopic dermatitis among young children in central and eastern Taiwan.

## Introduction

The increased prevalence and severity of allergic symptoms have become an important issue for the global disease burden.^1^ Approximately 20% of children and 3% of adults have been diagnosed with atopic dermatitis worldwide.^2^ A recent study found that adults with active or severe atopic dermatitis had an increased risk of all-cause death and certain causes of death, such as infectious and respiratory diseases.^3^ Atopic dermatitis begins to emerge at age 1 to 2 years^4^ and is regarded as the initial stage in a series of childhood allergic diseases, such as atopic asthma.^5^

The risk factors associated with atopic dermatitis include genetic factors, obesity, climate factors, and environmental pollutants.^2^ Previous studies have reported an association between exposure to heavy metals, including cadmium and lead and allergic diseases in adults.^6,7^ Another recent study found that prenatal inorganic arsenic exposure was associated with allergic airway inflammation in children up to age 14 years.^8^ Among infants, exposure to lead and chromium was associated with both atopic dermatitis symptoms and severity.^9^ However, the association between multiple metal exposure during pregnancy and the risk of atopic dermatitis in young children remains unknown.

Environmental exposures during gestational development might increase susceptibility to disease in later life.^10^ The mechanism of disease may involve activation of the aryl hydrocarbon receptor and the *NFE2L2* gene.^11^ Findings from studies of the general population have also suggested that allergic sensitization may be associated with increases in disease susceptibility.^12,13^ Fetal and early postnatal exposures to arsenic, cadmium, lead, mercury, and chromium may be important factors associated with immunotoxic effects.^14,15^ Because exposure to chemicals in the environment is complex and part of the exposome associated with health and disease,^16^ this birth cohort study aimed to investigate the association between prenatal exposure to multiple metals and the risk of atopic dermatitis in young children.

## Methods

### Study Population

**Figure 1.**
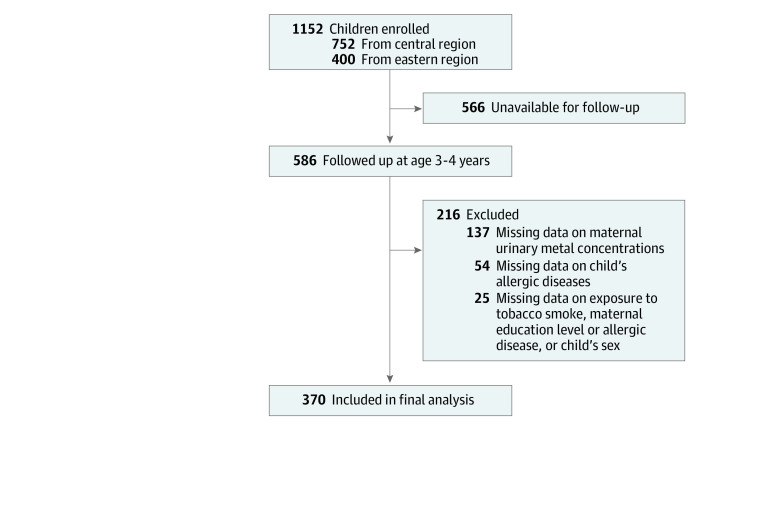
Flow Diagram of Participant Enrollment Process

The participants in this study were enrolled in the Taiwan Maternal and Infant Cohort Study (TMICS), a birth cohort study conducted at 9 hospitals in northern, central, southern, and eastern Taiwan. A total of 1152 pregnant women were enrolled in the initial TMICS from October 2012 to May 2015. Mothers and children from the central and eastern regions were later invited via telephone to participate in follow-up interviews conducted from August 2016 to January 2019. In total, 566 participants were unavailable for follow-up because they moved away from the area without leaving contact information or had scheduling conflicts, and 586 children were successfully followed up at age 4 years. A total of 216 children were excluded from the final analysis because they were missing data on (1) maternal urinary metal concentrations (n = 137); (2) allergic diseases in children at age 4 years (n = 54); or (3) exposure to environmental tobacco smoke among children at age 4 years, maternal educational level or allergic diseases, or child’s sex (n = 25). The final analysis of follow-up data included 370 mother and child pairs (Figure 1; eTable 1 in Supplement 1). The study was approved by the ethics committees of the National Health Research Institutes and the participating hospitals in Taiwan. All participating women provided written informed consent during their third trimester of pregnancy (for participation in the original study), and additional written informed consent was obtained before follow-up interviews. This study followed the Strengthening the Reporting of Observational Studies in Epidemiology (STROBE) reporting guideline for cohort studies.

### Data and Specimen Collection

Structured questionnaires, including items about demographic characteristics, lifestyle, dietary patterns, and residential environment, were administered to all participating women by trained interviewers during the third trimester of pregnancy. Physical measurements (eg, height, weight, and blood pressure) and urine samples were obtained, with urine samples collected in glass bottles and stored at −20 °C until analysis. Follow-up interviews were conducted in person at participants’ homes or participating hospitals. The presence of atopic dermatitis among children was defined as a positive response to the question, “Has your child ever had atopic dermatitis diagnosed by a physician?” during the questionnaire interview at age 4 years.

### Assessment of Metals in Maternal Urine

Frozen urinary specimens were moved to a 4 °C refrigerator for 2 to 4 hours, then defrosted at room temperature (26 °C-28 °C). Urinary metal concentrations were quantified using an inductively coupled plasma mass spectrometer (Agilent 7700x ICP-MS; Agilent Technologies) with a quadrupole mass filter and autosampler. For quality control, we used standard reference materials, spike recovery rate, and blank and duplicate samples. We performed the quality control regimen for every 10 samples, and the control spike recovery rates ranged from 89.0% to 114.5%. The limits of detection were for 0.399 μg/L^−1^ for arsenic, 0.066 μg/L^−1^ for cadmium, 0.016 μg/L^−1^ for lead, 0.225 μg/L^−1^ for cobalt, 0.090 μg/L^−1^ for copper, 0.022 μg/L^−1^ for nickel, 0.008 μg/L^−1^ for thallium, and 0.417 μg/L^−1^ for zinc. For instances in which the outcome was below the limit of detection, concentrations were estimated as the limit of detection divided by the square root of 2. Metal concentrations in maternal urine were divided by urine creatinine for urine volume correction (μg/g creatinine).

### Estimation of Total Inorganic Arsenic in Maternal Urine

The urinary concentration of inorganic arsenic represents the toxic form of total arsenic exposure, and the urinary concentration of arsenic species (arsenite, arsenate, monomethylarsonate, and dimethylarsenate) provides a better estimate of inorganic arsenic exposure than the concentration in blood. We calculated total inorganic arsenic as the sum of arsenite, arsenate, monomethylarsonate, and dimethylarsenate. We estimated total inorganic arsenic using a linear regression analysis of data from a previous TMICS pilot study^8^ derived from 336 women for whom both total urinary arsenic and total inorganic arsenic concentrations were available (with estimated total arsenic level calculated as the sum of 21.35241 and 0.14493 multiplied by the arsenic level).

Estimated total inorganic arsenic concentrations were highly correlated with total inorganic arsenic concentrations (*r* = 0.61; *P* < .001) (eTable 2 in Supplement 1). Therefore, we used estimated total inorganic arsenic when assessing total inorganic arsenic concentrations.

### Statistical Analysis

Mean values with SDs were used to summarize continuous variables, and 2-sided unpaired *t* tests were used to compare the differences between groups. Median values with IQRs, mean values with SDs, and Mann-Whitney *U* tests were used to summarize the concentrations of maternal urinary metals and compare the differences between groups. We used frequencies with percentages to summarize categorical variables and χ^2^ tests to comparison groups. Maternal urinary metal concentrations were log_2_-transformed for normality. Logistic regression analyses were used to estimate the association between prenatal exposure to 8 metals (arsenic, cadmium, lead, cobalt, copper, nickel, thallium, and zinc) and the risk of atopic dermatitis in children. We calculated *Q* values to assess the multiple comparison effect and reduce type 1 error probability.^17^

We adjusted for the child’s sex (male or female), the presence of parental allergies (yes or no), geographic area (central or eastern), exposure to tobacco smoke during pregnancy and among children at age 4 years (yes or no), and maternal educational level (<12 years, 13-16 years, or >16 years). In sensitivity analyses, we used a directed acyclic graph to select the minimum sufficient factor adjustment for the model.^18^ We also calculated propensity scores for potential imbalances in exposure groups, and we reduced bias by using a process resembling randomization.^19^ Previous studies have reported that parental allergies might be associated with 30% of the risk of asthma in children^20^ and the risk of atopic dermatitis in infants.^21^ In addition, maternal allergies may be associated with a higher risk of atopic dermatitis among children compared with paternal allergies.^22^ Therefore, we stratified groups by maternal history of allergic diseases (including allergic dermatitis, asthma, and rhinitis) and included only participants with complete data on maternal allergies.

In the present study, maternal urinary metal concentrations were highly correlated among metals (eFigure 1 in Supplement 1); thus, we performed a generalized weighted quantile sum (WQS) regression analysis to evaluate the mixture effect of coexposure to metals.^23^ The data were randomly divided into training (40%) and validation (60%) sets. We used a bootstrap method on the training data set to quantify the WQS index. The validation data set was then used to test the accuracy of the WQS index. The possible selection threshold of metals was defined as the weight of each metal that was greater than the quotient (0.125) of the total sum of weights in the WQS index (sum = 1) divided by the number of metals (n = 8). We used SAS software, version 9.4 (SAS Institute), to analyze the results of *t* tests, Mann-Whitney *U* tests, χ^2^ tests, and logistic regression analyses, and we used R software, version 4.0.2 (R Foundation for Statistical Computing), to calculate generalized WQS. A 2-sided *P* value of less than .05 was considered statistically significant. Data were analyzed from February 2 to August 12, 2021.

## Results

Among 370 children included in the final analysis, the mean (SD) age was 3.94 (0.59) years, and the mean (SD) gestational age at birth was 38.68 (1.14) weeks; 208 children (56.2%) were male, and 162 children (43.8%) were female. In total, 267 children (72.2%) were from the central area, and 103 children (27.8%) were from the eastern area (Table 1). A total of 110 children (29.7%) had atopic dermatitis at age 4 years. The differences between children who were included vs excluded (n = 782) from the analysis were significant for geographic area (eg, central region: 267 participants [72.2%] vs 485 participants [62.0%], respectively; *P* < .001), maternal educational level (eg, 13-16 years of education: 258 participants [69.7%] vs 411 participants [72.0]; *P* = .01), annual household income (eg, <$32 420: 173 participants [48.6%] vs 359 participants [68.0%]; *P* < .001), the presence of maternal allergies (172 participants [46.5%] vs 135 participants [24.1%]; *P* < .001), child’s age (mean [SD], 3.94 [0.59] years vs 3.71 [0.64] years; *P* < .001), maternal age (mean [SD], 32.52 [4.04] years vs 31.52 [5.09] years; *P* = .003), child’s gestational age at birth (mean [SD], 38.68 [1.14] weeks vs 38.41 [1.63] weeks; *P* = .04), and maternal urinary concentration of copper (mean [SD], 19.52 [11.83] μg/g creatinine vs 20.77 [10.63] μg/g creatinine; *P* = .004) (eTable 1 in Supplement 1).

**Table 1.** Characteristics of Children in Birth Cohort

Characteristic	No./total No. (%)		*P* value
	Atopic dermatitis (n = 110)	No atopic dermatitis (n = 260)	
Geographic area			
Central	79/110 (71.8)	188/260 (72.3)	.92
Eastern	31/110 (28.2)	72/260 (27.7)	
Sex			
Male	57/110 (51.8)	151/260 (58.1)	.27
Female	53 (48.2)	109/260 (41.9)	
Maternal educational level, y			
≤12	17/110 (15.5)	30/260 (11.5)	.004
13-16	64/110 (58.2)	194/260 (74.6)	
>16	29/110 (26.4)	36/260 (13.8)	
Annual household income, $^a^			
<32 420	55/108 (50.9)	118/248 (47.6)	.84
32 420-47 763	30/108 (27.8)	74/248 (29.8)	
>47 763	23/108 (21.3)	56/248 (22.6)	
Exposure to tobacco smoke			
Prenatal^b^			
Yes	43/101 (42.6)	87/225 (38.7)	.51
No	58/101 (57.4)	138/225 (61.3)	
At age 4 y			
Yes	15/110 (13.6)	61/260 (23.5)	.03
No	95/110 (86.4)	199/260 (76.5)	
Parental allergies			
Maternal			
Yes	58/110 (52.7)	114/260 (43.8)	.12
No	52/110 (47.3)	146/260 (56.2)	
Paternal^c^			
Yes	51/86 (59.3)	74/175 (42.3)	.01
No	35/86 (40.7)	101/175 (57.7)	
Age, mean (SD), y	3.91 (0.61)	3.96 (0.58)	.51
Birth weight, mean (SD), g^d^	3128 (343.7)	3126 (399.7)	.97
Maternal age, mean (SD), y^e^	32.18 (4.02)	32.68 (4.05)	.35
Gestational age at birth, mean (SD), wk^f^	38.65 (1.08)	38.70 (1.17)	.77
Maternal urinary metal concentrations during pregnancy, μg/g creatinine			
Arsenic			
Median (IQR)	65.92 (42.49-109.68)	50.20 (31.92-81.34)	<.001
Mean (SD)	95.20 (98.48)	73.97 (112.40)	
Estimated total inorganic arsenic^g^			
Median (IQR)	30.91 (27.51-37.25)	28.62 (25.97-33.14)	<.001
Mean (SD)	35.14 (14.27)	32.07 (16.29)	
Cadmium			
Median (IQR)	0.81 (0.59-1.18)	0.81 (0.54-1.21)	.64
Mean (SD)	1.04 (0.83)	1.05 (1.10)	
Lead			
Median (IQR)	1.34 (1.03-1.77)	1.38 (0.99-1.80)	.97
Mean (SD)	2.26 (7.93)	1.75 (3.61)	
Cobalt			
Median (IQR)	1.31 (0.92-2.06)	1.37 (0.82-2.14)	.72
Mean (SD)	1.49 (0.82	1.66 (1.22)	
Copper			
Median (IQR)	17.88 (14.47-20.85)	17.28 (14.14-21.77)	.66
Mean (SD)	19.09 (9.81)	19.70 (12.60)	
Nickel			
Median (IQR)	3.74 (2.46-5.75)	4.08 (2.40-6.80)	.37
Mean (SD)	6.07 (9.95)	6.92 (13.45)	
Thallium			
Median (IQR)	0.30 (0.23-0.38)	0.29 (0.23-0.40)	.91
Mean (SD)	0.33 (0.15)	0.33 (0.24)	
Zinc			
Median (IQR)	463.90 (332.25-621.19)	439.50 (326.38-655.59)	.94
Mean (SD)	517.90 (385.20)	532.90 (342.90)	

^a^ Data missing for 14 participants.

^b^ Data missing for 44 participants.

^c^ Data missing for 109 participants.

^d^ Data missing for 20 participants.

^e^ Data missing for 106 participants.

^f^ Data missing for 202 participants.

^g^ Calculated as the sum of 21.35241 and 0.14493 multiplied by the arsenic level.

Children from the central vs eastern region were older (mean [SD], 4.02 [0.61] years vs 3.74 [0.49] years, respectively; *P* < .001) and had higher annual household income (eg, >$47 763: 67 participants [26.3%] vs 12 participants [11.9%]; *P* = .01), lower exposure to tobacco smoke at age 4 years (47 participants [17.6%] vs 29 participants [28.2%]; *P* = .02), a slightly lower gestational age at birth (mean [SD], 38.46 [0.11] weeks vs 39.01 [1.11] weeks; *P* = .002), and higher maternal urinary concentrations of cadmium (mean [SD], 1.18 [1.13] μg/g creatinine vs 0.71 [0.52] μg/g creatinine; *P* < .001), lead (mean [SD], 1.87 [3.58] μg/g creatinine vs 2.02 [8.19] μg/g creatinine; *P* < .001), nickel (mean [SD], 7.68 [14.52] μg/g creatinine vs 4.03 [2.49] μg/g creatinine; *P* = .03), and zinc (mean [SD], 554.2 [376.2] μg/g creatinine vs 461.7 [286.5] μg/g creatinine; *P* = .004) (eTable 3 in Supplement 1). The maternal educational level was significantly different between children with vs without atopic dermatitis at age 4 years (eg, 13-16 years of education: 64 participants [58.2%] vs 194 participants [74.6%], respectively; *P* = .004) (Table 1). Children with vs without atopic dermatitis had significantly lower exposure to tobacco smoke at age 4 years (15 participants [13.6%] vs 61 participants [23.5%], respectively; *P* = .03), a higher incidence of paternal allergies (51 of 86 participants [59.3%] vs 74 of 175 participants [42.3%]; *P* = .01), and higher maternal urinary concentrations of arsenic (mean [SD], 95.20 [98.48] μg/g creatinine vs 73.97 [112.40] μg/g creatinine; *P* < .001) and estimated total inorganic arsenic (mean [SD], 35.14 [14.27] μg/g creatinine vs 32.07 [16.29] μg/g creatinine; *P* < .001).

### Prenatal Exposure to Metals

In the fully adjusted model, every doubled increase in maternal urinary estimated total inorganic arsenic was significantly associated with 2.42-fold (95% CI, 1.33-4.39; *P* = .003) higher odds of atopic dermatitis in children at age 4 years (Table 2). All other maternal urinary metal concentrations were not associated with higher odds of atopic dermatitis (eg, cadmium: odds ratio [OR], 1.24 [95% CI, 0.91-1.69; *P* = .27]; lead: OR, 1.00 [95% CI, 0.74-1.34; *P* = .99]). In the sensitivity analysis, a significant association was found between maternal urinary arsenic concentrations and estimated total inorganic arsenic concentrations and atopic dermatitis according to the directed acyclic graph (arsenic: OR, 1.41 [95% CI, 1.15-1.74; *P* = .001]; estimated total inorganic arsenic: OR, 2.14 [95% CI, 1.23-3.73; *P* = .01]) and propensity scores (arsenic: OR, 1.49 [95% CI, 1.20-1.85; *P* < .001]; estimated total inorganic arsenic: OR, 2.36 [95% CI, 1.33-4.19; *P* = .003]) (eTable 4 and eFigure 2 in Supplement 1). We obtained similar results when other metals were further adjusted (eTable 5 in Supplement 1) and when urinary metal concentrations were not stratified by urinary creatinine level (eTable 6 in Supplement 1).

**Table 2.** Association Between Prenatal Exposure to Metals and Atopic Dermatitis in Children^**a**^

Maternal urinary metal levels, μg/g creatinine^b^	Fully adjusted model, OR (95% CI)^c^	*P* value	*Q* value
Estimated total inorganic arsenic^d^	2.42 (1.33-4.39)	.003	0.01
Cadmium	1.24 (0.91-1.69)	.18	0.27
Lead	1.00 (0.74-1.34)	.99	0.99
Cobalt	1.00 (0.77-1.29)	.97	0.97
Copper	0.96 (0.63-1.46)	.85	0.85
Nickel	0.90 (0.77-1.06)	.22	0.27
Thallium	0.98 (0.65-1.48)	.93	0.93
Zinc	0.91 (0.68-1.24)	.56	0.59

Abbreviation: OR, odds ratio.

^a^ Results of logistic regression analysis comprising 370 participants.

^b^ The concentrations of metals were log_2_-transformed.

^c^ Adjusted for child’s sex (male or female), parental allergies (yes or no), geographic area (central or eastern), exposure to tobacco smoke during pregnancy (yes or no; 44 missing values), exposure to tobacco smoke at age 4 years (yes or no), and maternal educational level (≤12 years, 13-16 years, or >16 years).

^d^ Calculated as the sum of 21.35241 and 0.14493 multiplied by the arsenic level.

### Prenatal Coexposure to Metals

**Figure 2.**
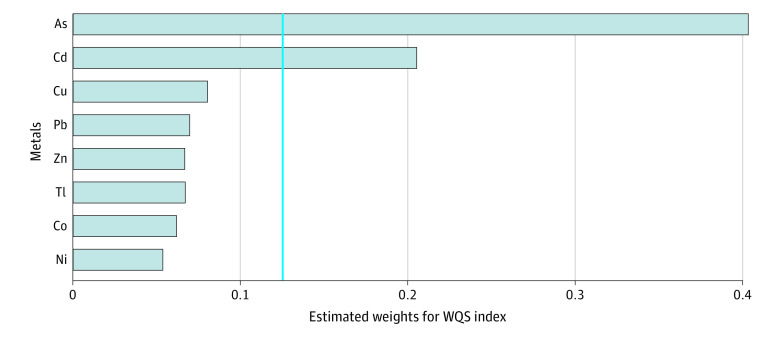
Estimated Weights of the Weighted Quantile Sum Index for Metals Prenatal coexposure to metals among children with atopic dermatitis at age 4 years. Adjusted for sex, maternal educational level, presence of parental allergies, exposure to tobacco smoke at age 4 years, and geographic area. The odds ratio for the risk of atopic dermatitis per unit increase in the derived weighted quantile sum (WQS) index of prenatal metal exposure was 1.63 (95% CI, 1.28-2.07). The solid blue line represents the boundary of the selection threshold. As indicates arsenic; Cd, cadmium; Co, cobalt; Cu, copper; Ni, nickel; Pb, lead; Tl, thallium; and Zn, zinc.

After adjustment for child’s sex, maternal educational level, parental allergies, exposure to tobacco smoke at age 4 years, and geographic area, every increase in unit in the derived generalized WQS index of maternal urinary metal concentrations was significantly associated with increased odds of atopic dermatitis (OR, 1.63; 95% CI, 1.28-2.07; *P* = .04). Arsenic (40.1%) and cadmium (20.5%) accounted for most of the WQS index (Figure 2).

### Maternal Allergies

Among children with mothers who had allergies, every doubled increase in maternal urinary arsenic concentrations and estimated total inorganic arsenic concentrations was significantly associated with increased odds of atopic dermatitis (OR, 1.61 [95% CI, 1.17-2.22; *P* = .003] and 3.51 [95% CI, 1.41-8.74; *P* = .01], respectively) after adjusting for sex, maternal educational level, geographic area, and exposure to tobacco smoke (Table 3). Similar results were obtained when we adjusted according to the directed acyclic graph (eg, estimated total inorganic arsenic: OR, 3.61 [95% CI, 1.47-8.85; *P* = .01]) and the sensitivity analysis (eg, estimated total inorganic arsenic: OR, 3.75 [95% CI, 1.50-9.35; *P* = .004]). For those without maternal allergies, no association was found between arsenic concentrations and atopic dermatitis (eg, estimated total inorganic arsenic: OR, 1.79 [95% CI, 0.84-3.80; *P* = .27]).

**Table 3.** Association Between Prenatal Exposure to Metals and Atopic Dermatitis in Children by Maternal Allergic Status^a^

Maternal urinary metal levels, μg/g creatinine^b^	OR (95%CI)	*P* value	*Q* value
**Model 1^c^**			
Maternal allergy			
Participants, No.	172	NA	NA
Estimated total inorganic arsenic^d^	3.51 (1.41-8.74)	.01	0.01
Cadmium	0.89 (0.58-1.37)	.60	0.60
Lead	1.25 (0.83-1.89)	.29	0.29
Cobalt	1.01 (0.71-1.46)	.95	0.95
Copper	0.80 (0.42-1.53)	.50	0.50
Nickel	0.84 (0.68-1.05)	.12	0.13
Thallium	1.09 (0.63-1.91)	.76	0.76
Zinc	0.85 (0.54-1.35)	.49	0.49
No maternal allergy			
Participants, No.	198	NA	NA
Estimated total inorganic arsenic^d^	1.79 (0.84-3.80)	.13	0.27
Cadmium	1.18 (0.81-1.73)	.39	0.63
Lead	0.88 (0.59-1.32)	.54	0.63
Cobalt	0.99 (0.72-1.37)	.97	0.97
Copper	1.20 (0.72-2.01)	.48	0.63
Nickel	0.99 (0.79-1.24)	.90	0.90
Thallium	0.98 (0.57-1.67)	.94	0.94
Zinc	0.99 (0.68-1.46)	.98	0.98
**Model 2^e^**			
Maternal allergy			
Participants, No.	172	NA	NA
Estimated total inorganic arsenic^d^	3.61 (1.47-8.85)	.01	0.01
Cadmium	0.94 (0.62-1.44)	.78	0.78
Lead	1.28 (0.86-1.90)	.23	0.29
Cobalt	1.01 (0.70-1.44)	.98	0.98
Copper	0.90 (0.48-1.66)	.72	0.72
Nickel	0.85 (0.68-1.05)	.13	0.19
Thallium	1.03 (0.60-1.78)	.91	0.91
Zinc	0.84 (0.53-1.33)	.46	0.49
No maternal allergy			
Participants, No.	198	NA	NA
Estimated total inorganic arsenic^d^	1.50 (0.74-3.06)	.26	0.46
Cadmium	1.11 (0.78-1.58)	.57	0.57
Lead	0.87 (0.58-1.28)	.47	0.56
Cobalt	0.99 (0.72-1.34)	.92	0.92
Copper	1.06 (0.66-1.70)	.80	0.80
Nickel	0.96 (0.78-1.19)	.72	0.72
Thallium	0.94 (0.58-1.53)	.80	0.80
Zinc	0.95 (0.66-1.37)	.79	0.79
**Sensitivity analysis^f^**			
Maternal allergy			
Participants, No.	172	NA	NA
Estimated total inorganic arsenic^d^	3.75 (1.50-9.35)	.004	<0.01
Cadmium	0.91 (0.59-1.39)	.65	0.65
Lead	1.39 (0.89-2.16)	.15	0.15
Cobalt	1.13 (0.73-1.73)	.58	0.58
Copper	0.80 (0.41-1.55)	.51	0.51
Nickel	0.79 (0.62-1.00)	.05	0.05
Thallium	0.95 (0.53-1.72)	.87	0.87
Zinc	0.81 (0.51-1.29)	.38	0.38
No maternal allergy			
Participants, No.	198	NA	NA
Estimated total inorganic arsenic^d^	1.62 (0.77-3.37)	.20	0.20
Cadmium	1.12 (0.76-1.66)	.57	0.57
Lead	0.83 (0.53-1.31)	.42	0.42
Cobalt	1.07 (0.73-1.59)	.72	0.71
Copper	1.13 (0.66-1.94)	.66	0.66
Nickel	1.01 (0.78-1.30)	.97	0.97
Thallium	1.22 (0.71-2.10)	.47	0.47
Zinc	0.90 (0.60-1.33)	.59	0.59

Abbreviations: NA, not applicable; OR, odds ratio.

^a^ Results of logistic regression analysis comprising 370 participants.

^b^ The concentrations of metals were log_2_-transformed.

^c^ Model 1 was adjusted for child’s sex (male or female), maternal educational level (≤12 years, 13-16 years, or >16 years), geographic area (central or eastern), and exposure to tobacco smoke at age 4 years (yes or no).

^d^ Calculated as the sum of 21.35241 and 0.14493 multiplied by the arsenic level.

^e^ Model 2 was adjusted for child’s sex (male or female) and geographic area (central or eastern) according to the directed acyclic graph.

^f^ The sensitivity analysis was adjusted for propensity score, which was calculated using child’s sex, exposure to tobacco smoke at age 4 years, maternal educational level, geographic area, and exposure to other metals (arsenic, cadmium, lead, cobalt, copper, nickel, thallium, and zinc).

## Discussion

This cohort study found that prenatal exposure to arsenic was positively associated with the risk of atopic dermatitis among children aged 4 years in Taiwan, and the odds of developing atopic dermatitis were approximately 2.4-fold higher when estimated total inorganic arsenic was evaluated. We also observed a significant association between estimated total inorganic arsenic concentrations and the development of atopic dermatitis among children who had mothers with allergies. The low exposure to tobacco smoke among children with atopic dermatitis in this study may be associated with altered behavior among parents attempting to protect their children with allergic diseases.

The pathophysiological features of atopic dermatitis include epidermal barrier dysfunction, skin inflammation, interaction between the nervous and immune systems, genetic susceptibility, and the effect of microbiota.^24^ The polarization of T cells in atopic dermatitis is considered biphasic, with a predominant response from helper T (T_H_) 2 cells in the acute phase and from T_H_1 cells in the chronic phase, and T_H_2 cells have been suggested to have important roles in cutaneous allergic inflammation as an integral part of atopic dermatitis.^25^ A previous study reported that newborns with the specific epigenetic pattern of T_H_2 cell-skewed immunity may have increased responses to common environmental allergens later in life.^26^ Exposure to arsenic during pregnancy has been associated with a DNA methylation pattern in infants^27^ as well as changes in children’s immunoglobulin E levels.^28^

Although metals other than arsenic (ie, cadmium, lead, cobalt, copper, nickel, thallium, and zinc) were examined in this study, these metals were not associated with the incidence of atopic dermatitis in children at age 4 years; however, an association was found between prenatal coexposure to these metals (including arsenic) and atopic dermatitis in children. The exposure to cadmium was secondary to arsenic in the WQS index. Cadmium is also a well-established toxic metal that can detrimentally impact many organs, including the bones, kidneys, and pancreas.^29^ Kim et al^30^ observed an association between prenatal cadmium exposure and atopic dermatitis in infants aged 6 months, and elevated cadmium concentrations in cord blood were associated with an increased risk of atopic dermatitis.

Hanson et al^31^ treated pregnant mice with 10 ppm of cadmium chloride as an environmentally relevant dose. They found that cadmium exposure may induce decreases in the production of interferon gamma and CD8^+^CD223^+^ T cells in the spleens of their offspring at age 7 weeks. A recent study reported that cadmium concentrations mediated by in-cell viability had differentially functional effects on in vitro murine macrophages and mast cells after cadmium exposure.^32^ Mast cells and macrophages have been suggested to play a role in atopic dermatitis–associated inflammation and pruritus. For example, interleukin 31 is derived from T_H_2 cells, macrophages, mast cells, and mast cells in atopic dermatitis lesions with higher interleukin 31 immunoreactivity than normal skin.^33^ Interleukin 23, secreted by dendritic cells and macrophages, has been documented to be an important cytokine to control inflammation in peripheral tissues. Skin tissues of patients with atopic dermatitis have substantially increased activation of the T_H_17/interleukin 23 and T_H_22 axes and keratinocyte production.^34^ Thus, prenatal exposure to cadmium may be associated with atopic dermatitis in early childhood through disruption of immune cell development in the fetus.

After stratifying the analysis by maternal history of allergic diseases, we also found a significant association between maternal arsenic exposure and atopic dermatitis in children at age 4 years. One plausible explanation for this association may be heritability, including genetic and epigenetic factors. Children with mothers who had allergies had greater odds of developing atopic dermatitis, which suggests a genetic role and an association with the in utero environment, which could shape neonatal immunity and, consequently, the development of atopic dermatitis in early childhood.^35^

The main sources of exposure to metals are diet, air pollution, or cigarette smoking (active or passive) among the general population, and pregnant women are particularly susceptible to metal exposure.^36,37,38^ Therefore, improving the environment and reducing contact with these potential sources are important steps to reducing the risk of exposure to metals in this susceptible population.

### Strengths and Limitations

This study has strengths. Its main strength is its birth cohort design, in which urinary samples were collected from pregnant women in the third trimester and used to estimate the likelihood that children would develop atopic dermatitis 3 or 4 years later. The use of urine sampling could have increased the participation rate among pregnant women because urine sampling is less invasive than venous blood sampling. Moreover, blood arsenic concentrations only reflect recent exposure and cannot be used as a reliable estimate of chronic exposure.^39^ Our participants are children aged 4 years, and atopic dermatitis is the most commonly observed allergy that can be well diagnosed. The results were similar to those obtained when the analysis was adjusted for the presence of different urinary metals at age 4 years (data not shown), probably owing to the different exposure scenarios. We used well-designed methods for data collection. We examined the association between coexposure to a mixture of metals and atopic dermatitis because of the complex consequences of metal exposure for human health, and some urinary metal concentrations were highly correlated.^40^

This study also has limitations. First, there may be insufficient power in the statistical analyses because of the relatively small sample. However, the bootstrap method used in the generalized WQS model was applied to the training data set to reduce the partial effects of insufficient power. Second, we collected maternal urine samples in glass bottles; thus, we only measured total arsenic concentration and did not separately measure the concentrations of individual arsenic species. Therefore, total inorganic arsenic values were not measured directly but estimated using an equation derived from data on total arsenic and total inorganic arsenic concentrations from a previous birth cohort study among pregnant women residing in the same geographic area. Two basic metabolic processes of inorganic arsenic are oxidation-reduction and methylation reactions. Approximately 70% of absorbed organic and inorganic arsenic is excreted from the kidneys via urine.^41^

Third, we used parent-reported atopic dermatitis in this study without obtaining data from a complete clinical examination conducted in parallel by a physician. The reliance on parental reporting might produce underestimation of the results through nondifferential misclassification because the women did not have knowledge of their urinary metal concentrations in the third trimester; thus, they would not have been likely to overreport their children’s atopic dermatitis (vs people who may overreport symptoms or disease when they realize that they have had high exposure). Moreover, the parent-reported presence of atopic dermatitis in children was based on the question, “Has your child ever had atopic dermatitis diagnosed by a physician?” This question may be more reliable than, “Has your child ever had atopic dermatitis?”

Fourth, we reported observations among a birth cohort of children aged 4 years residing in the central and eastern regions of Taiwan; thus, external validity may be low, and results may not be generalizable to all children aged 4 years in Taiwan. Fifth, the possibility of residual or unmeasured confounding from genetic factors and exposure to other environmental chemicals cannot be eliminated. Epigenetic factors, such as modifications to genomic DNA and posttranscriptional micro-RNA regulation, have also been associated with the development of atopic dermatitis.^42^ In addition, we adjusted the model for the presence of parental allergies and further stratified the analysis by maternal allergic status to reduce partial confounding from genetic factors.

## Conclusions

This birth cohort study found an association between prenatal coexposure to metals and atopic dermatitis in children at age 4 years. Arsenic followed by cadmium accounted for most of the metal mixture index associated with atopic dermatitis. Prevention of exposure to inorganic arsenic and cadmium during pregnancy may be helpful for the control of atopic dermatitis and other potential allergic diseases in young children. Environmental improvements and reductions in contact with sources of exposure are important to reduce the risk of metal exposure among this susceptible population.

## Notes

Supplement 1. **eTable 1.** Baseline Characteristics of Children Included, Unavailable for Follow-up, and Excluded **eTable 2.** Spearman Correlation Coefficients for Total Arsenic, Total Inorganic Arsenic, and Estimated Total Inorganic Arsenic in Maternal Urine During Pregnancy in Previous TMICS Pilot Study With Arsenic Data Available **eTable 3.** Characteristics of Children in Birth Cohort Classified by Geographic Area **eTable 4.** Logistic Regression Analysis of Association Between Prenatal Exposure to Metals and Atopic Dermatitis in Children **eTable 5.** Logistic Regression Analysis of Association Between Prenatal Exposure to Metals and Atopic Dermatitis in Children With Further Adjustment for Metals **eTable 6.** Logistic Regression Analysis of Association Between Prenatal Exposure to Metals and Atopic Dermatitis in Children Without Categorization by Creatinine Urinary Levels **eFigure 1.** Correlation Matrix of Maternal Urinary Metal Concentrations **eFigure 2.** Directed Acyclic Graph of Confounding Variables

Supplement 2. **Nonauthor Collaborators.** TMICS Study Group
