# Errors in Methods

**DOI:** 10.1001/jamanetworkopen.2021.48833

**Published:** 2022-02-07

In the Original Investigation titled “Association Between Prenatal Exposure to Metals and Atopic Dermatitis Among Children Aged 4 Years in Taiwan,”^1^ published October 27, 2021, data errors have been corrected in the Methods section. Under “Assessment of Metals in Maternal Urine,” the sentence on the limits of detection now reads as follows: “The limits of detection were 0.399 μg/L for arsenic, 0.066 μg/L for cadmium, 0.022 μg/L for lead, 0.016 μg/L for cobalt, 0.225 μg/L for copper, 0.090 μg/L for nickel, 0.008 μg/L for thallium, and 0.417 μg/L for zinc.” This article has been corrected.^1^
