# Supplementary material for: Association Between Prenatal Exposure to Metals and Atopic Dermatitis Among Children Aged 4 Years in Taiwan
Source: JAMA Netw Open. 2021 Oct 27;4(10):e2131327. doi: 10.1001/jamanetworkopen.2021.31327 (PMC8552055; doi:10.1001/jamanetworkopen.2021.31327)
Supplement: Supplement 1. — eTable 1. Baseline Characteristics of Children Included, Unavailable for Follow-up, and Excluded eTable 2. Spearman Correlation Coefficients for Total Arsenic, Total Inorganic Arsenic, and Estimated Total Inorganic Arsenic in Maternal Urine During Pregnancy in Previous TMICS Pilot Study With Arsenic Data Available eTable 3. Characteristics of Children in Birth Cohort Classified by Geographic Area eTable 4. Logistic Regression Analysis of Association Between Prenatal Exposure to Metals and Atopic Dermatitis in Children eTable 5. Logistic Regression Analysis of Association Between Prenatal Exposure to Metals and Atopic Dermatitis in Children With Further Adjustment for Metals eTable 6. Logistic Regression Analysis of Association Between Prenatal Exposure to Metals and Atopic Dermatitis in Children Without Categorization by Creatinine Urinary Levels eFigure 1. Correlation Matrix of Maternal Urinary Metal Concentrations eFigure 2. Directed Acyclic Graph of Confounding Variables [file jamanetwopen-e2131327-s001.pdf]

Supplementary Online Content

Tsai TL, Wang SL, Hsieh CJ, et al; TMICS Study Group. Association between prenatal exposure to metals and atopic dermatitis among children aged 4 years in Taiwan. *JAMA Netw Open*. 2021;4(10):e2131327. doi:10.1001/jamanetworkopen.2021.31327

eTable 1. Baseline Characteristics of Children Included, Unavailable for Follow-up, and Excluded

eTable 2. Spearman Correlation Coefficients for Total Arsenic, Total Inorganic Arsenic, and Estimated Total Inorganic Arsenic in Maternal Urine During Pregnancy in Previous TMICS Pilot Study With Arsenic Data Available

eTable 3. Characteristics of Children in Birth Cohort Classified by Geographic Area

eTable 4. Logistic Regression Analysis of Association Between Prenatal Exposure to Metals and Atopic Dermatitis in Children

eTable 5. Logistic Regression Analysis of Association Between Prenatal Exposure to Metals and Atopic Dermatitis in Children With Further Adjustment for Metals

eTable 6. Logistic Regression Analysis of Association Between Prenatal Exposure to Metals and Atopic Dermatitis in Children Without Categorization by Creatinine Urinary Levels

eFigure 1. Correlation Matrix of Maternal Urinary Metal Concentrations

eFigure 2. Directed Acyclic Graph of Confounding Variables

This supplementary material has been provided by the authors to give readers additional information about their work.

© 2021 Tsai TL et al. *JAMA Network Open*.

eTable 1. Baseline Characteristics of Children Included, Unavailable for Follow-up, and Excluded (N = 1152)

		Lost to follow-up or Excluded (n=782)		Included (n=370)		p-value
		n (%)		n (%)		
Geographic area	Central	485 (62.02)		267 (72.16)		< 0.01
	Eastern	297 (37.98)		103 (27.84)		
Sex	Boy	297 (50.94)		208 (56.22)		0.11
	Girl	286 (49.06)		162 (43.78)		
Maternal education (yrs)	≤ 12	95 (16.64)		47 (12.70)		0.01
	13–16	411 (71.98)		258 (69.73)		
	> 16	65 (11.38)		65 (17.57)		
Income per year (USD)	< 32420	359 (67.99)		173 (48.60)		< 0.01
	32420–47763	148 (28.03)		104 (29.21)		
	> 47763	21 (3.98)		79 (22.19)		
ETS exposure at pregnancy	Yes	256 (44.91)		130 (39.88)		0.14
	No	314 (55.09)		196 (60.12)		
Maternal allergies	Yes	135 (24.11)		172 (46.49)		< 0.01
	No	425 (75.89)		198 (53.51)		
		Mean ± SD		Mean ± SD		
Age		3.71 ± 0.64		3.94 ± 0.59		< 0.01
Birth weight (g)		3080 ± 449.3		3126 ± 383.4		0.11
Maternal age		31.52 ± 5.09		32.52 ± 4.04		< 0.01
Gestational weeks		38.41 ± 1.63		38.68 ± 1.14		0.04
		Median (IQR)	Mean ± SD	Median (IQR)	Mean ± SD	
$U_{maternal}$ metal levels (μg·g cre^{-1})	As	50.27 (50.87)	69.93 ± 70.20	53.72 (53.03)	80.28 ± 108.7	0.07
	eΣiAs[§]	28.64 (7.37)	31.48 ± 10.17	29.14 (7.69)	32.98 ± 15.76	0.07
	Cd	0.80 (0.51)	0.98 ± 0.81	0.81 (0.64)	1.05 ± 1.02	0.32
	Pb	1.31 (0.85)	1.48 ± 1.10	1.35 (0.78)	1.90 ± 5.27	0.29
	Co	1.27 (1.00)	1.60 ± 1.33	1.33 (1.30)	1.61 ± 1.12	0.24
	Cu	18.63 (8.84)	20.77 ± 10.63	17.44 (7.48)	19.52 ± 11.83	< 0.01
	Ni	3.98 (4.00)	6.16 ± 11.90	3.95 (3.97)	6.67 ± 12.51	0.85
	Tl	0.31 (0.17)	0.33 ± 0.16	0.29 (0.16)	0.33 ± 0.22	0.56
	Zn	439.3 (337.2)	498.4 ± 311.4	453.6 (322.8)	528.4 ± 355.5	0.14

[§] eΣiAs (estimated total inorganic arsenic) = 21.35241 + 0.14493*(As)

© 2021 Tsai TL et al. *JAMA Network Open*.

eTable 2. Spearman Correlation Coefficients for Total Arsenic, Total Inorganic Arsenic, and Estimated Total Inorganic Arsenic in Maternal Urine During Pregnancy in Previous TMICS Pilot Study With Arsenic Data Available (N = 336)

Species (μg·g cre^{-1})	As	ΣiAs	eΣiAs§
As	1	0.61	1.00
		< 0.01	< 0.01
ΣiAs		1	0.61
			< 0.01
eΣiAs§			1

ΣiAs (total inorganic arsenic) = As(III) + As(V) + MMA + DMA
§ eΣiAs (estimated total inorganic arsenic) = 21.35241 + 0.14493*(As)
The concentration of ΣiAs (median: 32.80; IQR: 22.50)
The concentration of eΣiAs (median: 34.08; IQR: 13.68)

© 2021 Tsai TL et al. *JAMA Network Open*.

eTable 3. Characteristics of Children in Birth Cohort Classified by Geographic Area

		**Central (n=267)**		**Eastern (n=103)**		**p-value**
		n (%)		n (%)		
Sex	Boy	156 (58.43)		52 (50.49)		0.17
	Girl	111 (41.57)		51 (49.51)		
Maternal education (yrs)	≤ 12	29 (10.86)		18 (17.48)		0.15
	13–16	193 (72.28)		65 (63.11)		
	> 16	45 (16.85)		20 (19.42)		
Income per year (USD)	< 32420	115 (45.10)		58 (57.43)		0.01
	32420–47763	73 (28.63)		31 (30.69)		
	> 47763	67 (26.27)		12 (11.88)		
ETS exposure at pregnancy	Yes	88 (38.43)		42 (43.30)		0.46
	No	141 (61.57)		55 (56.70)		
ETS exposure at 4 yrs	Yes	47 (17.60)		29 (28.16)		0.02
	No	220 (82.40)		74 (71.84)		
Paternal allergies	Yes	93 (47.21)		32 (50.00)		0.70
	No	104 (52.79)		32 (50.00)		
Maternal allergies	Yes	127 (47.57)		45 (43.69)		0.50
	No	140 (52.43)		58 (56.31)		
Parental allergies	Yes	170 (63.67)		58 (56.31)		0.19
	No	97 (36.33)		45 (43.69)		
AD	Yes	79 (29.59)		31 (30.10)		0.92
		Mean ± SD		Mean ± SD		
Age		4.02 ± 0.61		3.74 ± 0.49		< 0.01
Birth weight (g)		3128 ± 383.0		3123 ± 386.6		0.92
Maternal age		32.62 ± 3.99		32.35 ± 4.15		0.61
Gestational weeks		38.46 ± 0.11		39.01 ± 1.11		< 0.01
		Median (IQR)	Mean ± SD	Median (IQR)	Mean ± SD	
$U_{maternal}$ metal levels (μg·g cre^{-1})	As	57.30 (51.28)	81.75 ± 119.7	48.94 (68.36)	76.47 ± 7.38	0.39
	eΣiAs[§]	29.66 (7.43)	33.20 ± 17.35	28.45 (9.91)	32.44 ± 10.63	0.39
	Cd	0.92 (0.71)	1.18 ± 1.13	0.61 (0.33)	0.71 ± 0.52	< 0.01
	Pb	1.44 (0.75)	1.87 ± 3.58	1.11 (0.67)	2.02 ± 8.19	< 0.01
	Co	1.37 (1.31)	1.63 ± 1.15	1.29 (1.24)	1.55 ± 1.05	0.50
	Cu	17.33 (6.96)	19.67 ± 12.21	18.33 (8.43)	19.14 ± 10.82	0.83
	Ni	4.09 (4.87)	7.68 ± 14.52	3.60 (3.08)	4.03 ± 2.49	0.03
	Tl	0.30 (0.16)	0.34 ± 0.24	0.29 (0.17)	0.31 ± 0.13	0.18
	Zn	468.5 (316.9)	554.2 ± 376.2	389.3 (307.7)	461.7 ± 286.5	< 0.01

[§] eΣiAs (estimated total inorganic arsenic) = 21.35241 + 0.14493*(As)

© 2021 Tsai TL et al. *JAMA Network Open*.

eTable 4. Logistic Regression Analysis of Association Between Prenatal Exposure to Metals and Atopic Dermatitis in Children (N = 370)

	Model 1			Model 2		
$U_{maternal}$ **metal levels (μg·g cre^{-1})**	**OR (95%CI)**	**p-value**	**q value**	**OR (95%CI)**	**p-value**	**q value**
As	1.41 (1.15, 1.74)	< 0.01	0.01	1.49 (1.20, 1.85)	< 0.01	< 0.01
eΣiAs[§]	2.14 (1.23, 3.73)	0.01	0.02	2.36 (1.33, 4.19)	< 0.01	0.01
Cd	1.04 (0.80, 1.36)	0.74	0.98	1.02 (0.76, 1.36)	0.90	0.90
Pb	1.05 (0.80, 1.37)	0.74	0.97	1.06 (0.79, 1.41)	0.72	0.72
Co	0.99 (0.79, 1.25)	0.93	0.98	1.11 (0.83, 1.47)	0.49	0.51
Cu	1.01 (0.69, 1.45)	0.98	0.98	1.00 (0.67, 1.50)	0.99	0.99
Ni	0.91 (0.79, 1.06)	0.23	0.47	0.88 (0.74, 1.04)	0.14	0.21
Tl	1.03 (0.72, 1.48)	0.86	0.98	1.04 (0.70, 1.53)	0.86	0.86
Zn	0.94 (0.72, 1.24)	0.66	0.98	0.83 (0.62, 1.12)	0.23	0.28

Model 1: Adjustment for child's sex (boy or girl), geographic area (central or eastern) according to DAG
Model 2: Adjustment for propensity score for each exposure. It was calculated from child's sex (boy or girl), ETS exposure at 4 years of age (yes or no), maternal education (≤ 12 yrs, 13–16 yrs, > 16 yrs), parental allergies (yes or no), geographic area (central or eastern), exposure to other metals (the studied metal did not include)
[§] eΣiAs (estimated total inorganic arsenic) = 21.35241 + 0.14493*(As)
The concentrations of metals were log_2-transformed

© 2021 Tsai TL et al. *JAMA Network Open*.

eTable 5. Logistic Regression Analysis of Association Between Prenatal Exposure to Metals and Atopic Dermatitis in Children With Further Adjustment for Metals (N = 370)

	Model 1		Model 1		Model 2		Model 2	
**$U_{maternal}$ metal levels ($\mu g \cdot g$ cre^{-1})**	**OR (95%CI)**	**p-value**	**OR (95%CI)**	**p-value**	**OR (95%CI)**	**p-value**	**OR (95%CI)**	**p-value**
As	1.48 (1.19, 1.84)	< 0.01	—		1.47 (1.18, 1.84)	< 0.01	—	
eΣiAs§	—		2.34 (1.31, 4.19)	< 0.01	—		2.28 (1.27, 4.09)	0.01
Cd	1.01 (0.74, 1.37)	0.97	1.02 (0.75, 1.39)	0.89	1.02 (0.75, 1.40)	0.90	1.03 (0.76, 1.41)	0.83
Pb	1.01 (0.74, 1.39)	0.94	1.03 (0.75, 1.40)	0.88	1.03 (0.74, 1.43)	0.86	1.04 (0.76, 1.44)	0.79
Co	1.07 (0.81, 1.43)	0.62	1.08 (0.81, 1.43)	0.60	1.07 (0.80, 1.43)	0.63	1.08 (0.81, 1.44)	0.61
Cu	1.06 (0.68, 1.66)	0.78	1.06 (0.68, 1.64)	0.21	1.12 (0.71, 1.78)	0.62	1.12 (0.71, 1.77)	0.62
Ni	0.87 (0.73, 1.04)	0.12	0.87 (0.73, 1.03)	0.11	0.86 (0.72, 1.03)	0.10	0.86 (0.72, 1.03)	0.10
Tl	0.92 (0.61, 1.40)	0.70	0.94 (0.62, 1.42)	0.77	0.87 (0.56, 1.34)	0.52	0.89 (0.58, 1.36)	0.58
Zn	0.84 (0.62, 1.14)	0.26	0.87 (0.64, 1.17)	0.35	0.80 (0.58, 1.10)	0.17	0.83 (0.60, 1.13)	0.23

§ eΣiAs (estimated total inorganic arsenic) = 21.35241 + 0.14493*(As)
Model 1: Adjustment for child's sex (boy or girl), geographic area (central or eastern) according to DAG; all metals in the model
Model 2: Adjustment for child's sex (boy or girl), parental allergies (yes or no), geographic area (central or eastern), ETS exposure at 4 years of age (yes or no); all metals in the model
The concentrations of metals were $\log_2$-transformed

© 2021 Tsai TL et al. *JAMA Network Open*.

eTable 6. Logistic Regression Analysis of Association Between Prenatal Exposure to Metals and Atopic Dermatitis in Children Without Categorization by Creatinine Urinary Levels (N = 370)

	Model 1			Model 2		
**$U_{maternal}$ metal levels (µg/L)**	**OR (95%CI)**	**p-value**	**q value**	**OR (95%CI)**	**p-value**	**q value**
As	1.32 (1.12, 1.56)	< 0.01	0.01	1.32 (1.11, 1.57)	< 0.01	0.01
eΣiAs[§]	2.25 (1.25, 4.05)	0.01	0.03	2.22 (1.23, 4.02)	0.01	0.04
Cd	1.08 (0.91, 1.30)	0.38	0.63	1.10 (0.91, 1.32)	0.32	0.55
Pb	1.12 (0.91, 1.38)	0.30	0.63	1.14 (0.92, 1.41)	0.24	0.55
Co	1.06 (0.88, 1.27)	0.54	0.63	1.07 (0.89, 1.29)	0.50	0.64
Cu	1.08 (0.88, 1.31)	0.46	0.63	1.09 (0.90, 1.34)	0.36	0.55
Ni	0.96 (0.84, 1.10)	0.56	0.63	0.97 (0.84, 1.10)	0.60	0.67
Tl	1.11 (0.89, 1.38)	0.36	0.63	1.11 (0.89, 1.39)	0.37	0.55
Zn	1.04 (0.86, 1.25)	0.69	0.69	1.04 (0.86, 1.26)	0.71	0.71

[§] eΣiAs (estimated total inorganic arsenic) = 15.065 + 0.122*(As)
Model 1: Adjustment for child's sex (boy or girl), geographic area (central or eastern) according to DAG; $U_{maternal}$ metal levels without urinary creatinine correction
Model 2: Adjustment for child's sex (boy or girl), parental allergies (yes or no), geographic area (central or eastern), ETS exposure at 4 years of age (yes or no); $U_{maternal}$ metal levels without urinary creatinine correction
The concentrations of metals were log_2-transformed

© 2021 Tsai TL et al. *JAMA Network Open*.

eFigure 1. Correlation Matrix of Maternal Urinary Metal Concentrations

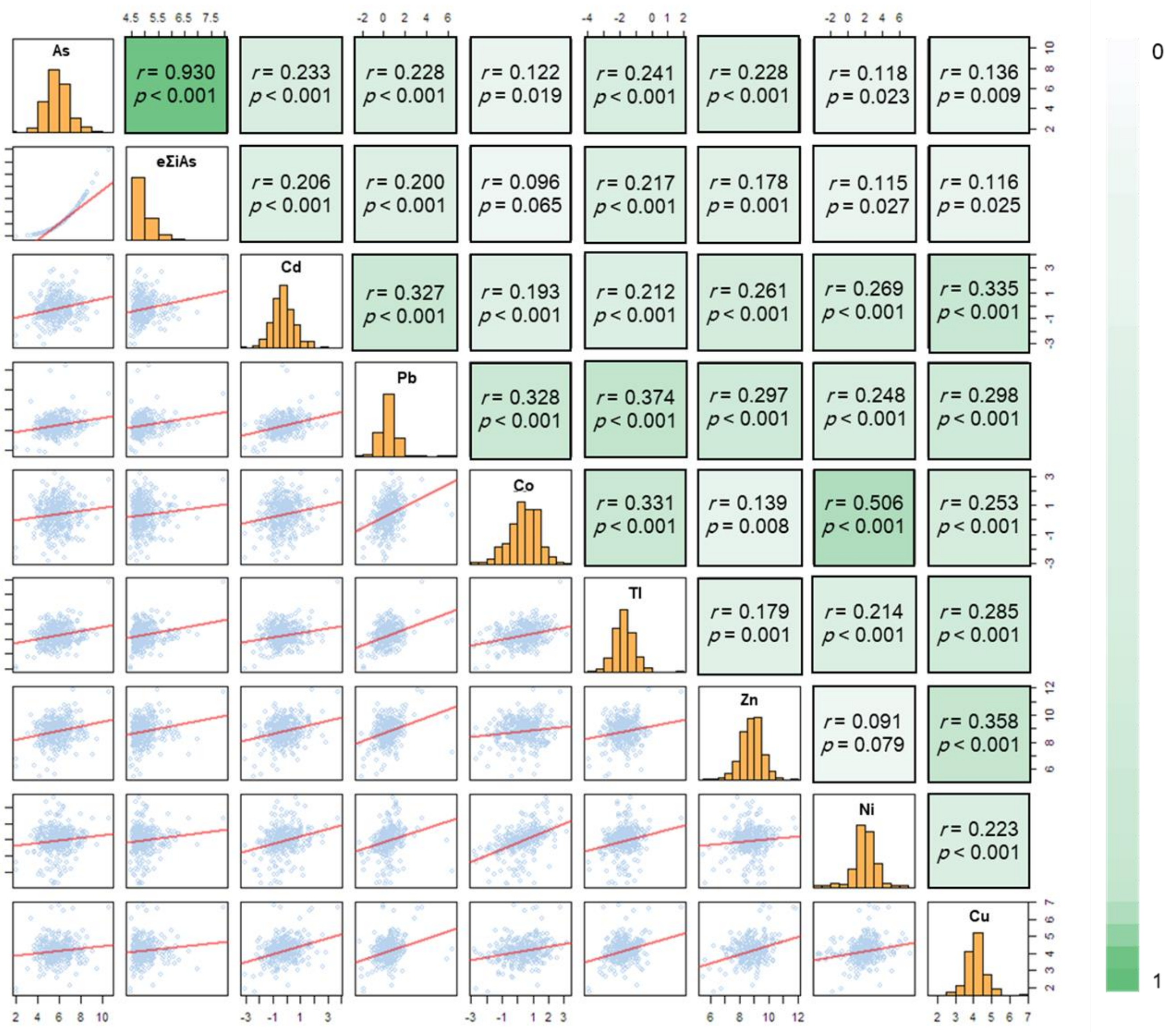

Metal concentrations measured by µg·g cre^{-1}. eΣiAs (estimated total inorganic arsenic) = 21.35241 + 0.14493*(As). The concentrations of metals were log_2-transformed for normality.

© 2021 Tsai TL et al. *JAMA Network Open*.

eFigure 2. Directed Acyclic Graph of Confounding Variables

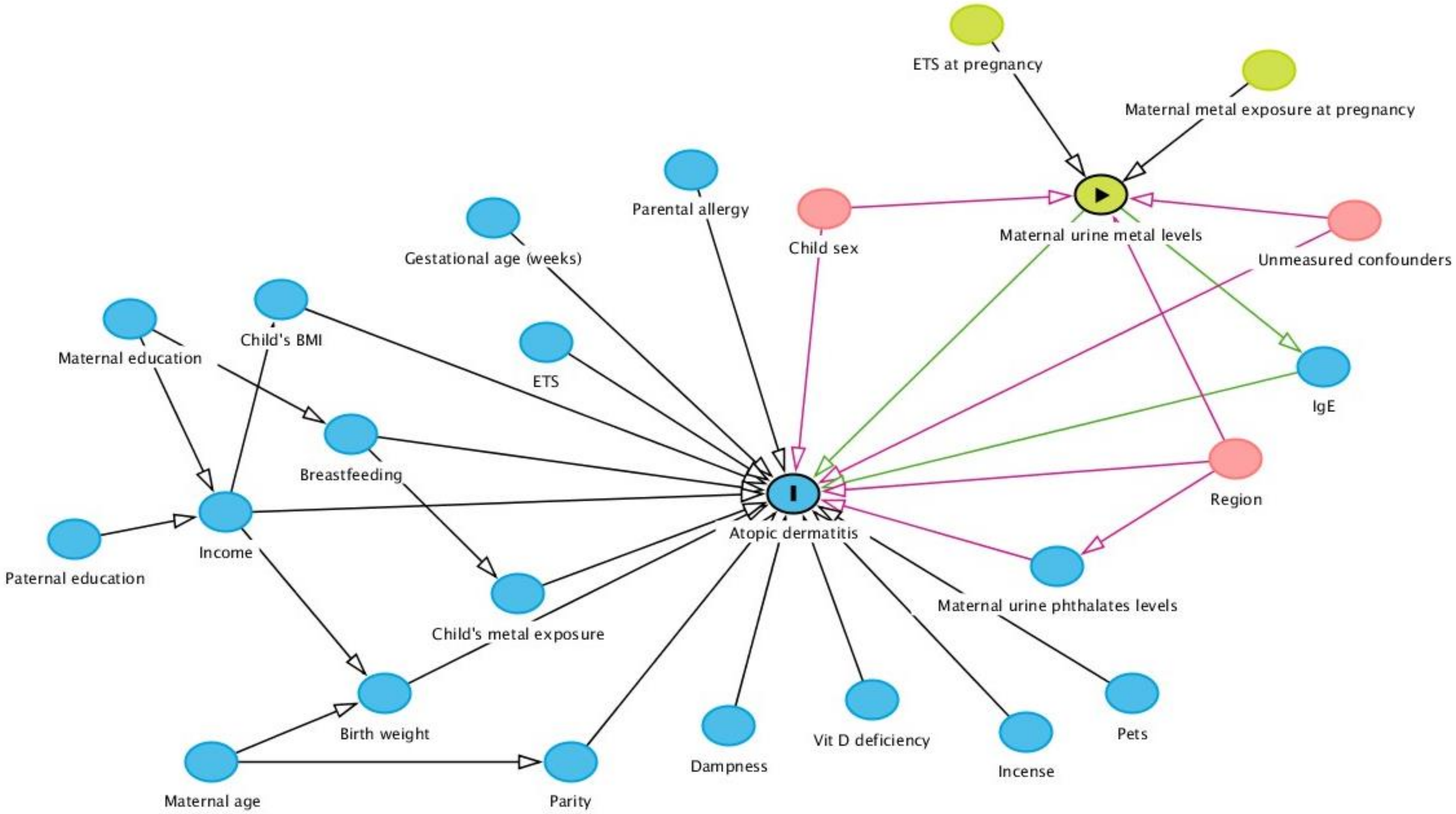

Red oval: confounding factor; blue oval: other factors; green oval: ancestor of exposure; green oval with solid triangle: exposure; blue oval with vertical bar: outcome.

© 2021 Tsai TL et al. *JAMA Network Open*.
