# Supplementary material for: Association Between Prenatal Exposure to Metals and Atopic Dermatitis Among Children Aged 4 Years in Taiwan
Source: JAMA Netw Open. 2021 Oct 27;4(10):e2131327. doi: 10.1001/jamanetworkopen.2021.31327 (PMC8552055; doi:10.1001/jamanetworkopen.2021.31327)
Supplement: Supplement 2. — Nonauthor Collaborators. TMICS Study Group [file jamanetwopen-e2131327-s002.pdf]

Supplemental Online Content: Nonauthor Collaborators

*Indicates required information. Only first name, last name, and suffix will appear in PubMed.

| *Group Name(s): TMICS Study Group | | | | | | | |
|---|---|---|---|---|---|---|---|
| ***First Name and Middle Initial(s)** | ***Last Name** | ***Suffix (eg, Jr, III)** | Academic Degrees | Institution | Location (city, state/province, country) | Role or Contribution, eg, chair, principal investigator | Group (if more than 1 Group listed in the byline) and/or Subgroup (eg, Steering Committee) |
| Ying- Chih | Tseng | | MD | Hsinchu Cathay General Hospital | Hsinchu, Taiwan | Cooperative physician; subject recruitments | TMICS-Central area |
| Chaw-Liang | Chang | | MD | Hsinchu Cathay General Hospital | Hsinchu, Taiwan | Cooperative physician; subject recruitments | TMICS-Central area |
| Tsung-Ho | Ying | | MD | Chung Shan Medical University Hosp | Taichung, Taiwan | Cooperative physician; subject recruitments | TMICS-Central area |
| Pen-Hua | Su | | MD | Chung Shan Medical University Hosp | Taichung, Taiwan | Cooperative physician; subject recruitments | TMICS-Central area |
| Kuei-Cheng | Hsu | | MD | Changhua Christian Hospital | Changhua, Taiwan | Cooperative physician; subject recruitments | TMICS-Central area |
| Hsiao-Neng | Chen | | MD | Changhua Christian Hospital | Changhua, Taiwan | Cooperative physician; subject recruitments | TMICS-Central area |
| Jian-Wun | Cian | | MD | Changhua Christian Hospital | Changhua, Taiwan | Cooperative physician; subject recruitments | TMICS-Central area |
| Wei-Te | Lei | | MD | Hsinchu Mackay Memorial Hospital | Hsinchu, Taiwan | Cooperative physician; subject recruitments | TMICS-Central area |
| Sheng-Po | Kao | | MD | Buddhist Tzu Chi General Hospital | Hualien, Taiwan | Cooperative physician; subject recruitments | TMICS-Eastern area |

*Indicates required information. Only first name, last name, and suffix will appear in PubMed.

| ***First Name and Middle Initial(s)** | ***Last Name** | ***Suffix (eg, Jr, III)** | Academic Degrees | Institution | Location (city, state/province, country) | Role or Contribution, eg, chair, principal investigator | Group (if more than 1 Group listed in the byline) and/or Subgroup (eg, Steering Committee) |
|---|---|---|---|---|---|---|---|
| Yu-Chi | Wei | | MD | Buddhist Tzu Chi General Hospital | Hualien, Taiwan | Cooperative physician; subject recruitments | TMICS-Eastern area |
| Yu-Hsun | Chang | | MD | Buddhist Tzu Chi General Hospital | Hualien, Taiwan | Cooperative physician; subject recruitments | TMICS-Eastern area |
| Fu-Chen | Kuo | | MD | E-Da Hospital | Kaohsiung, Taiwan | Cooperative physician; subject recruitments | TMICS-Southern area |
| Chien-Yi | Wu | | MD | E-Da Hospital | Kaohsiung, Taiwan | Cooperative physician; subject recruitments | TMICS-Southern area |
